# Abundant intratumoral fibrosis prevents lymphocyte infiltration into peritoneal metastases of colorectal cancer

**DOI:** 10.1371/journal.pone.0255049

**Published:** 2021-07-22

**Authors:** En Wang, Masatsune Shibutani, Hisashi Nagahara, Tatsunari Fukuoka, Yasuhito Iseki, Yuki Okazaki, Shinichiro Kashiwagi, Hiroaki Tanaka, Kiyoshi Maeda, Kosei Hirakawa, Masaichi Ohira

**Affiliations:** 1 Department of Gastroenterological Surgery, Osaka City University Graduate School of Medicine, Abeno-ku, Osaka, Japan; 2 Department of Breast and Endocrine Surgery, Osaka City University Graduate School of Medicine, Abeno-ku, Osaka, Japan; 3 Department of Gastroenterological Surgery, Osaka City General Hospital, Miyakojima-ku, Osaka, Japan; University of Alabama at Birmingham, UNITED STATES

## Abstract

**Background:**

Tumor-infiltrating lymphocytes (TILs) have been reported to reflect the anti-tumor immune status. However, recent investigations have demonstrated that intratumoral fibrosis is important as a factor affecting the infiltration of TILs. This study investigated the organ specificities of TIL infiltration and intratumoral fibrosis in primary colorectal cancer and distant metastases, as well as the relationship between the distribution of TILs and intratumoral fibrosis.

**Methods:**

Patients who underwent resection of primary tumors or distant metastases for colorectal cancer with distant metastases were enrolled. We evaluated the TIL infiltration by immunohistochemical staining with CD3&CD8 and intratumoral fibrosis by immunohistochemical staining with α-SMA positive cancer-associated fibroblasts and Masson’s trichrome staining against collagen fibers. The "ImageJ" was used to evaluate fibrosis, and the density of TILs in the dense and sparse areas of fibrosis was calculated. The Immunoscore (IS) was obtained based on the density of CD3^+^/CD8^+^TILs in the tumor center and invasive margin of the primary tumor.

**Results:**

The degree of CD3^+^/CD8^+^TIL infiltration in peritoneal metastases was significantly lower than that in liver and lung metastases. The area ratio of α-SMA positive cancer-associated fibroblasts and collagen fibers in peritoneal metastases was significantly higher than that of liver and lung metastases. Furthermore, the density of TILs in the high-fibrosis area was significantly lower than that in the low-fibrosis area. In the high-IS group of primary tumors, the degree of TIL infiltration in distant metastases was significantly higher than that in the low-IS group.

**Conclusion:**

The infiltration of T lymphocytes into tumors is prevented in peritoneal metastases of colorectal cancer due to the high intratumoral fibrosis, which may lead to treatment resistance and a poor prognosis.

## Introduction

Colorectal cancer was the third-most common cancer worldwide in 2018 after lung and breast cancer, with the second highest mortality rate after lung cancer [1]. Colorectal peritoneal metastasis is considered a non-curative factor that contributes to a poor prognosis, representing the second-most common location for synchronous distant metastasis after the liver [2, 3] and the fourth-most common site of recurrence after curative surgery [3, 4]. In recent years, the prognosis of peritoneal metastasis has been improved with advances in surgical techniques, anticancer drugs, molecular-targeted drugs, and immunotherapy, but it remains poor compared to that of lung and liver metastases [2, 3, 5–7]. Therefore, peritoneal metastasis is subclassified as the most advanced stage IVC / IVc under the TNM classification of malignant tumors and the Japanese classification of colorectal, appendiceal and anal carcinoma [8, 9].

Previous studies have revealed that the tumor microenvironment, including immune cells, extracellular matrix, and cytokines, plays an important role in tumor initiation, proliferation, invasion, and metastasis [10–12]. Among the immune effector cells in the tumor, tumor-infiltrating lymphocytes (TILs) reportedly reflect the anti-tumor immune status of the host and correlate with the prognosis and therapeutic outcomes of various cancers, including colorectal cancer [10, 11, 13–16]. However, most studies on TILs have focused on primary tumors, and few have focused on TILs in metastases, the situation of which is poorly understood.

Recent studies have suggested that, like primary tumors, the degree of TIL infiltration in the metastases may correlate with the prognosis and therapeutic outcomes [17–22]. It has also been gradually clarified that the degree of TIL infiltration in the metastases varies with the metastatic organ [18, 19, 23]. In previous studies, the mechanism underlying the difference of TILs in each metastatic organ was unclear, but recent studies have suggested that TIL infiltration may relate to suppressive immune cells, neovascular dysfunction, suppression by chemokines and cytokines, and intratumoral fibrosis [11, 24, 25].

In recent years, intratumoral fibrosis has been focused on as a factor playing an important role in the infiltration of immunocompetent cells [11, 24, 26, 27]. Although intratumoral fibrosis in peritoneal metastases has been reported to be very abundant [28, 29], there have been few studies on local immunity in peritoneal metastases. Peritoneal metastases are well known to have treatment resistance and be associated with a poor prognosis, ostensibly because the infiltration of immunocompetent cells is suppressed by intratumoral fibrosis.

In the present study, we investigated the local immune status and intratumoral fibrosis in peritoneal metastases of colorectal cancer and compared the findings among distant metastases. The local immune status in the distant metastases has been suggested to correlate with the prognosis and therapeutic outcomes, similar to the situation in primary tumors, but the relationship between the local immune status in the primary tumors and that in distant metastases has been poorly understood. We therefore compared the local immune status between primary tumors and distant metastases, including peritoneal metastases.

## Methods

### 1. Patients

A total of 218 patients who underwent resection of primary tumors or distant metastases for colorectal cancer with distant metastases at the department of Surgery, Osaka City University (Osaka, Japan) between January 1994 and December 2019, including 67 cases of peritoneal metastases, 138 cases of liver metastases, 25 cases of lung metastases and 160 cases of primary tumors of metastases (58 cases without primary tumors), were enrolled.

This study was performed in compliance with the principles expressed in the Declaration of Helsinki and was approved by the ethics committee of Osaka City University (approved no. 3853).

### 2. Methods

Surgical specimens treated with formalin fixative and paraffin embedding were used to prepare 4-μm-thick sections, and all sections were immunohistochemically stained and subjected to Masson’s trichrome staining (MTS). CD3, a pan-T lymphocytes marker that plays a central role in antitumor immunity, and CD8, a cytotoxic T lymphocyte marker, were used for the evaluation of TIL infiltration, while cancer-related fibroblasts (CAFs) and collagen fibers, which are the main components of fibrosis, were used for the evaluation of fibrosis in the tumor microenvironment. α-SMA was used as the marker of CAF, and collagen fibers were evaluated using the blue-stained area on MTS, with collagen fibers stained blue.

#### Immunohistochemistry

Sections were deparaffined and rehydrated and then subjected to endogenous peroxidase blocking in 1% H_2_O_2_ solution in methanol for 15 minutes. Antigen retrieval was carried out by autoclaving the sections at 105°C for 10 minutes each in Dako Target Retrieval Solution (Dako, Glostrup, Denmark). Serum blocking was carried out with antibody in 10% normal rabbit serum (Nichirei Biosciences, Tokyo, Japan) for 10 minutes. After H_2_O_2_ and serum blocking, the slides were incubated with primary monoclonal mouse anti-human antibody at room temperature for 1 h or at 4°C overnight. The secondary antibody was biotin-labeled rabbit anti-mouse IgG, IgA, and IgM (1:500; Nichirei Biosciences). Detection was performed with a DAB kit (Histofine simple stain kit; Nichirei Biosciences). The sections were counterstained with hematoxylin. Anti-CD3 antibody (1:400 dilution; clone F7.2.38; Dako), anti-CD8 antibody (1:200 dilution; clone C8/144B, Dako), and anti-α-SMA antibody (1:300 dilution; clone 1A4; Dako) were used as primary antibodies.

Specimens of lymph nodes containing T lymphocytes were used as a positive control for immunohistochemical staining of anti-CD3 and anti-CD8. The α-SMA expression in vascular smooth muscle was used as an internal positive control for immunohistochemical staining of anti-α-SMA.

#### MTS

MTS was performed using a Masson Staining Kit (Muto Pure Chemicals, Tokyo, Japan) for tumor tissue samples according to the manufacturer’s protocol. All sections were deparaffined, rehydrated, and then washed in distilled water for 5 minutes, re-fixed in the first mordant solution for 20 minutes, rinsed with running warm tap water for 3 minutes, stained with Weigert’s iron hematoxylin solution for 10 minutes, rinsed with running warm tap water for 5 minutes, re-fixed in the second mordant solution for 30 seconds, rinsed with running warm tap water for 1 minute, stained with 0.75% orange solution for 1 minute; stained with Masson stain B solution for 20 minutes, stained with 2.5% phosphotungstic acid solution for 15 minutes; stained with aniline blue for 5 minutes, dehydrated, cleared with xylene, and mounted with marinol.

#### Double staining by immunohistochemical staining and MTS

First, immunohistochemical staining of anti-CD3 and CD8 was performed until the detection step with a DAB kit, and then washing in distilled water was performed without counterstaining for hematoxylin. MTS was then performed using the above-mentioned protocol.

#### The immunohistochemical evaluation for TILs

The method established in a previous study performed in our laboratory was used to evaluate the local immunity of distant metastases [30]. The process is briefly described below. The inner part of the tumor margin of the liver and lung metastases was observed as the target area. The specimens of peritoneal metastases were small, and the margin of some specimens had been crushed, so the central part was observed as the target area. The target areas of the tumors were identified at a low magnification with an optical microscope (BX53, Olympus, Tokyo, Japan), and then the number of TILs was counted in a randomly selected field of the target area at a magnification of 400x. The mean values obtained in five different fields was used for the data analysis.

The local immunity of the primary tumor was evaluated using the Immunoscore, which sensitively reflected the antitumor immune status of the host and could be evaluated based on the density of CD3^+^ and CD8^+^ TILs.

#### Determination of the Immunoscore

The Immunoscore of the primary tumor was calculated by a modified method based on the Immunoscore evaluation method reported in previous studies [31–35]. The outline is shown below. The Immunoscore was defined as a scoring system based on the combination of two lymphocyte populations with markers of CD3 and CD8 in two regions, including the invasive margin (IM) and tumor center (CT). The number of CD3^+^/CD8^+^ TILs was counted in a randomly selected field at a magnification of 400x, and the mean values obtained in five different fields of each region were defined as the density of CD3^+^/CD8^+^ TIL in these regions. We obtained four values for the density of TILs for each tumor: CD3^+^TILs in the IM, CD3^+^TIL in the CT, CD8^+^TILs in the IM, and CD8^+^TIL in the CT. The median density of TILs with each marker in each region was set as the cut-off value. According to the cut-off value, each tumor was classified as either having a high or low density of each marker in each region, and the total number of high densities observed in each tumor was defined as the Immunoscore of the tumor, ranging from 0 to 4. Cases with an Immunoscore 0–1 were defined as the low Immunoscore (low-IS) group, and those with an Immunoscore 2–4 were defined as the high Immunoscore (high-IS) group.

#### The evaluation of intratumoral fibrosis

**Fig 1:**
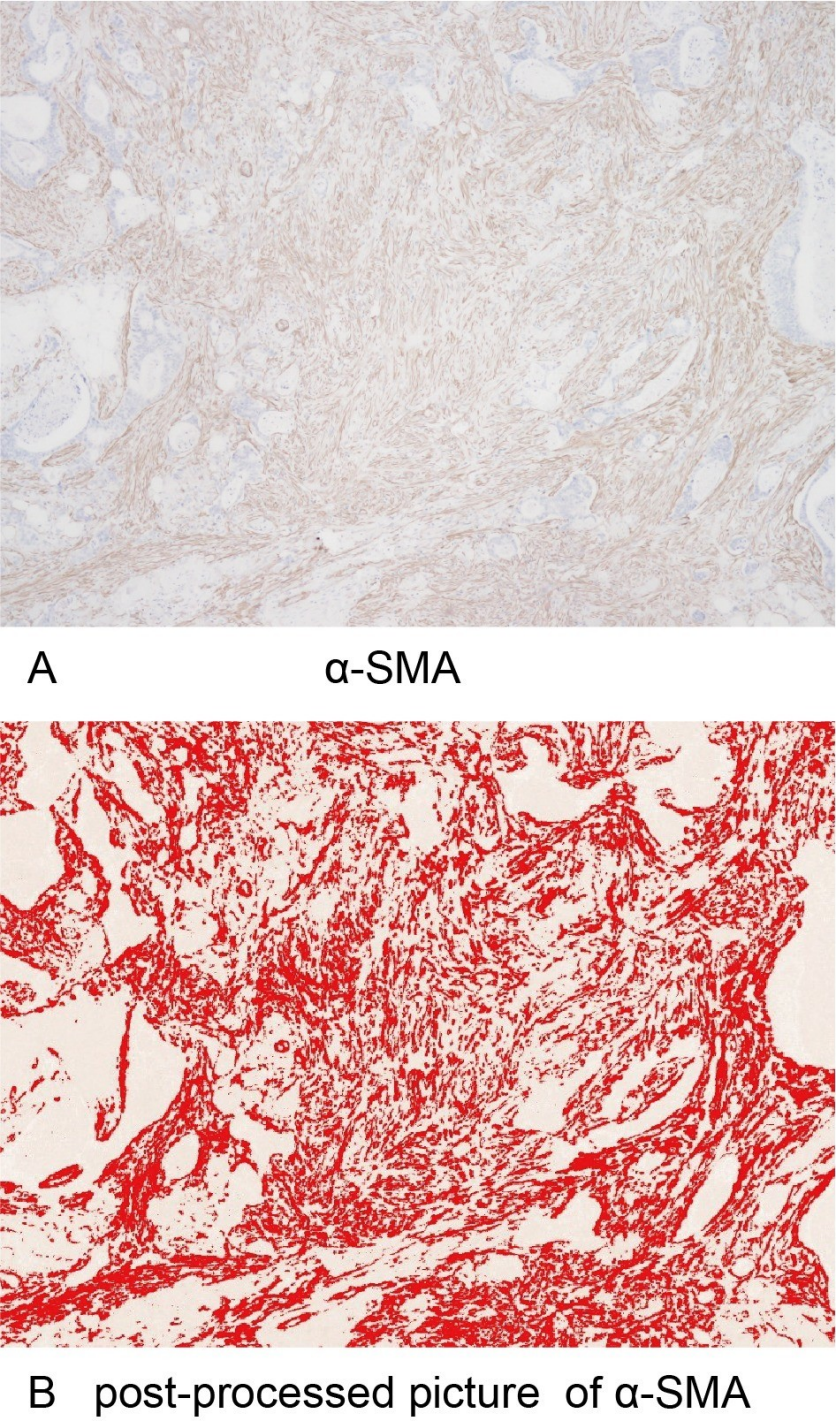
Original and post-processed images of α-SMA-positive cancer-associated fibroblasts. (A) α-SMA-positive cancer-associated fibroblasts at x40 HPF. (B) A post-processed image in which the brown area of α-SMA-positive cancer-associated fibroblasts was changed to a red color using ImageJ.

**Fig 2:**
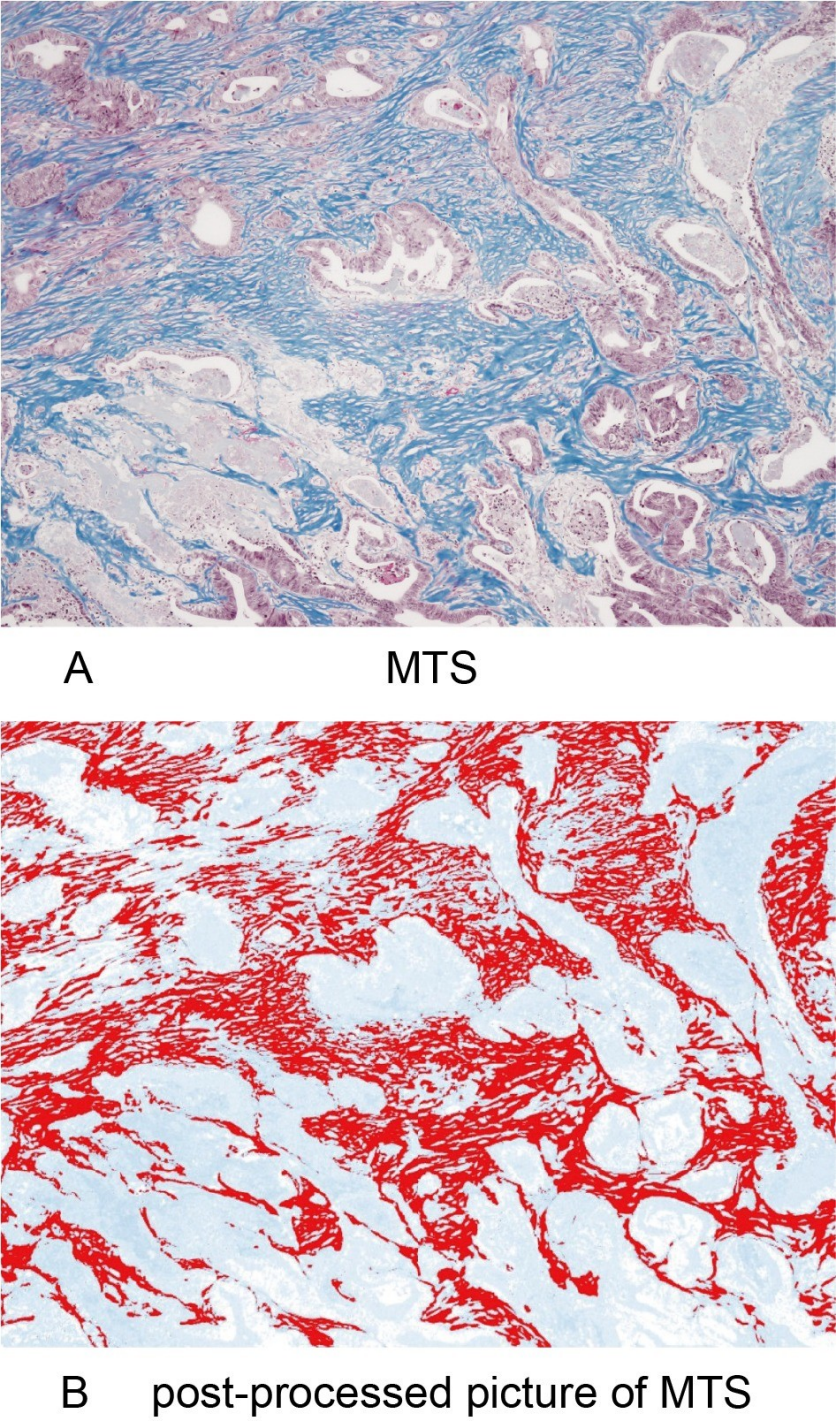
Original and post-processed images of Masson’s trichrome staining. (A) Collagen fibers stained blue on Masson’s trichrome staining at x40 HPF. (B) A post-processed image in which the blue area of collagen fibers was changed to a red color using ImageJ.

The same areas evaluated for TILs were observed as target areas for the evaluation of intratumoral fibrosis. The target areas of tumors were identified at a low magnification with an optical microscope (Olympus BX53), and then the images of CAFs and collagen fibers were collected from a randomly selected field of the target areas at a magnification of 40x. The area ratios of α-SMA^+^ CAF **(Fig 1)** and the blue-stained area (collagen fibers) **(Fig 2)** on MTS were determined using the image analysis software program "ImageJ" (version 1.52p, National Institutes of Health, Bethesda, MD, USA) with the plugin “color deconvolution” for stain separation [36, 37]. A yellow image was separated from the image of α-SMA^+^ CAF using the separation function for hematoxylin & DAB (H DAB) of the plugin, and a blue image was separated from the image of MTS using the separation function for MTS. The separated images were processed with a certain threshold value, the target areas were selected, and the area ratios of the target areas were obtained by the analyze function. The mean of values obtained in three different areas was used for the data analysis.

#### The evaluation of the relationship between the distribution of TILs and intratumoral fibrosis

**Fig 3:**
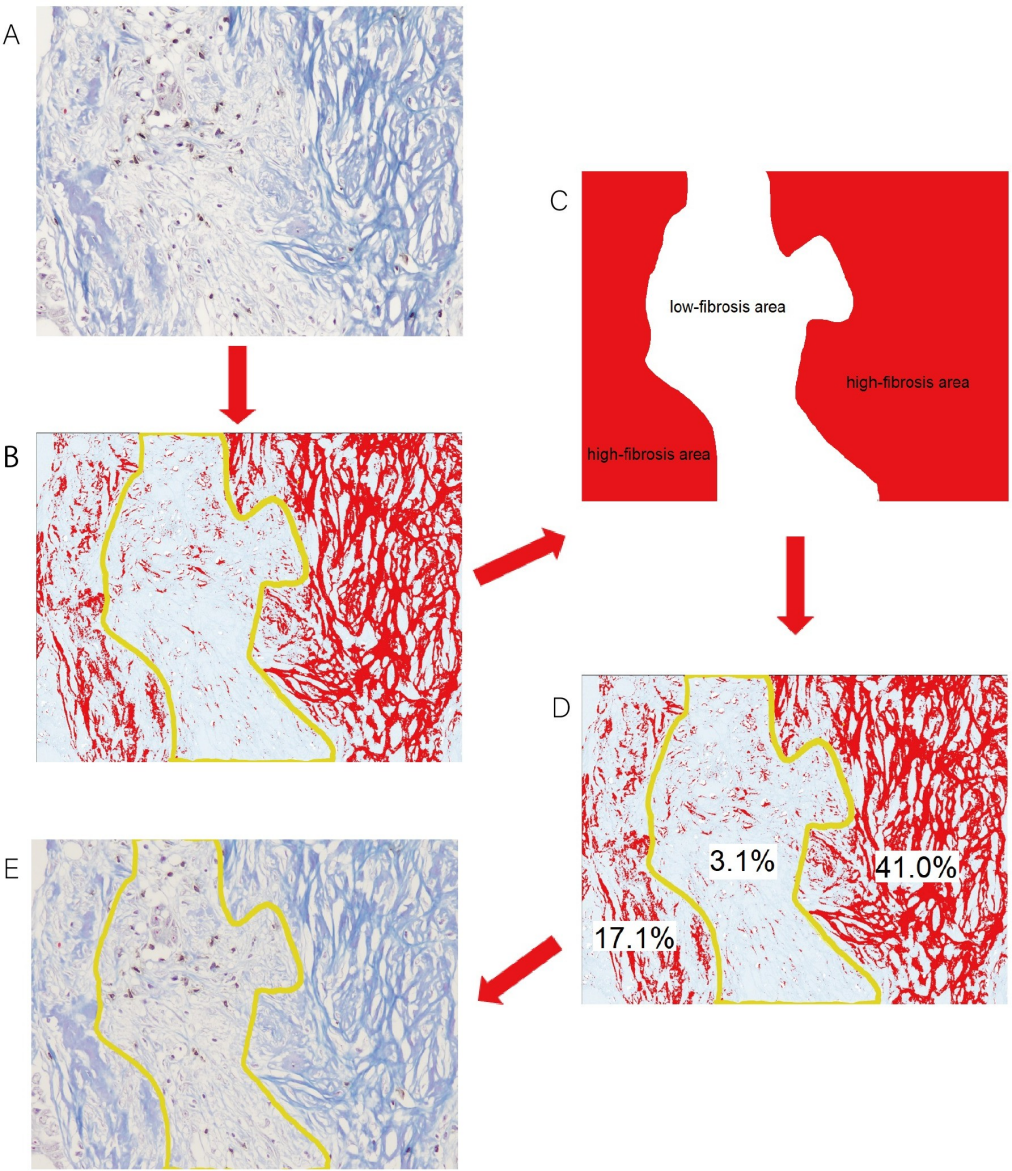
Regarding the evaluation of the distribution of TILs in the high-fibrosis area and that in the low-fibrosis area, the median value of the area ratios of collagen fibers in peritoneal metastases was defined as the cut-off value (15.64%). Areas with an area ratio of collagen fibers greater than the cut-off value (≥15.64%) were defined as high-fibrosis areas, and those with an area ratio of collagen fibers lower than the cut-off value (<15.64%) were defined as low-fibrosis areas. (A) The distribution of tumor-infiltrating lymphocytes in fibrotic areas on double-staining using immunohistochemical staining of anti-CD3 and anti-CD8 and Masson’s trichrome staining at x200 HPF. (B) A post-processed image in which the blue area of collagen fibers changed to a red color and were able to be manually separated into high- and low-fibrosis areas (C) according to the density of collagen fibers. (D) To verify the accuracy of the division, the area ratios of collagen fibers were calculated in each area using the ImageJ software program (area ratios of high-fibrosis areas of a sample image: 41.0% and 17.1%; area ratio of a low-fibrosis area of a sample image: 3.1%) and contrasted with the definitions. If inaccurate division was suspected, the division of the fibrosis area selected by the ImageJ software program was adjusted until the defined conditions were met. (E) The area ratios of the high- and low-fibrosis areas were calculated using the ImageJ software program, and the numbers of tumor-infiltrating lymphocytes in the high- and low-fibrosis areas were counted. The densities of CD3^+^/CD8^+^TILs in the high- and low-fibrosis areas were then calculated.

All sections of peritoneal metastasis that were double-stained with anti-CD3/anti-CD8 immunohistochemical staining and MTS were observed with an optical microscope (Olympus BX53). The field that included both high- and low-fibrosis areas and was relatively rich in TILs was selected for observation at a magnification of 200x. The median area ratio for the collagen fibers in peritoneal metastases was defined as the cut-off value. Areas with an area ratio of collagen fibers greater than the cut-off value were defined as high-fibrosis areas, while those with an area ratio of collagen fibers less than the cut-off value were defined as low-fibrosis areas. The area of collagen fibers in the double-stained images was selected using the ImageJ software program. The selected collagen fibers were manually divided into high- and low-fibrosis areas, according to the area ratios of collagen fibers, and the area ratios of the high- and low-fibrosis areas were calculated. To verify the accuracy of the division, the area ratios of collagen fibers were measured in each area using the ImageJ software program and contrasted with the definitions. If an inaccurate division was deemed to have been made, the division of the high- and low-fibrosis areas selected by the ImageJ software program was adjusted until the defined conditions were met. The number of CD3^+^TILs and CD8^+^TILs for each area was counted **(Fig 3)**. The mean of values obtained in three different fields was used for the data analysis, and the densities of CD3^+^/CD8^+^TILs in the high- and low-fibrosis areas were calculated.

### 3. Statistical analyses

Analyses were performed using the JMP 13 software program (SAS Institute, Cary, NC, USA). Differences in the CD3, CD8, α-SMA, and collagen fiber values between groups were analyzed using a two-sided Wilcoxon’s rank sum test, and differences in the density of TILs between the high- and low-fibrosis areas were analyzed using a two-sided paired *t*-test. Statistical significance was defined as p<0.05.

## Results

**Fig 4:**
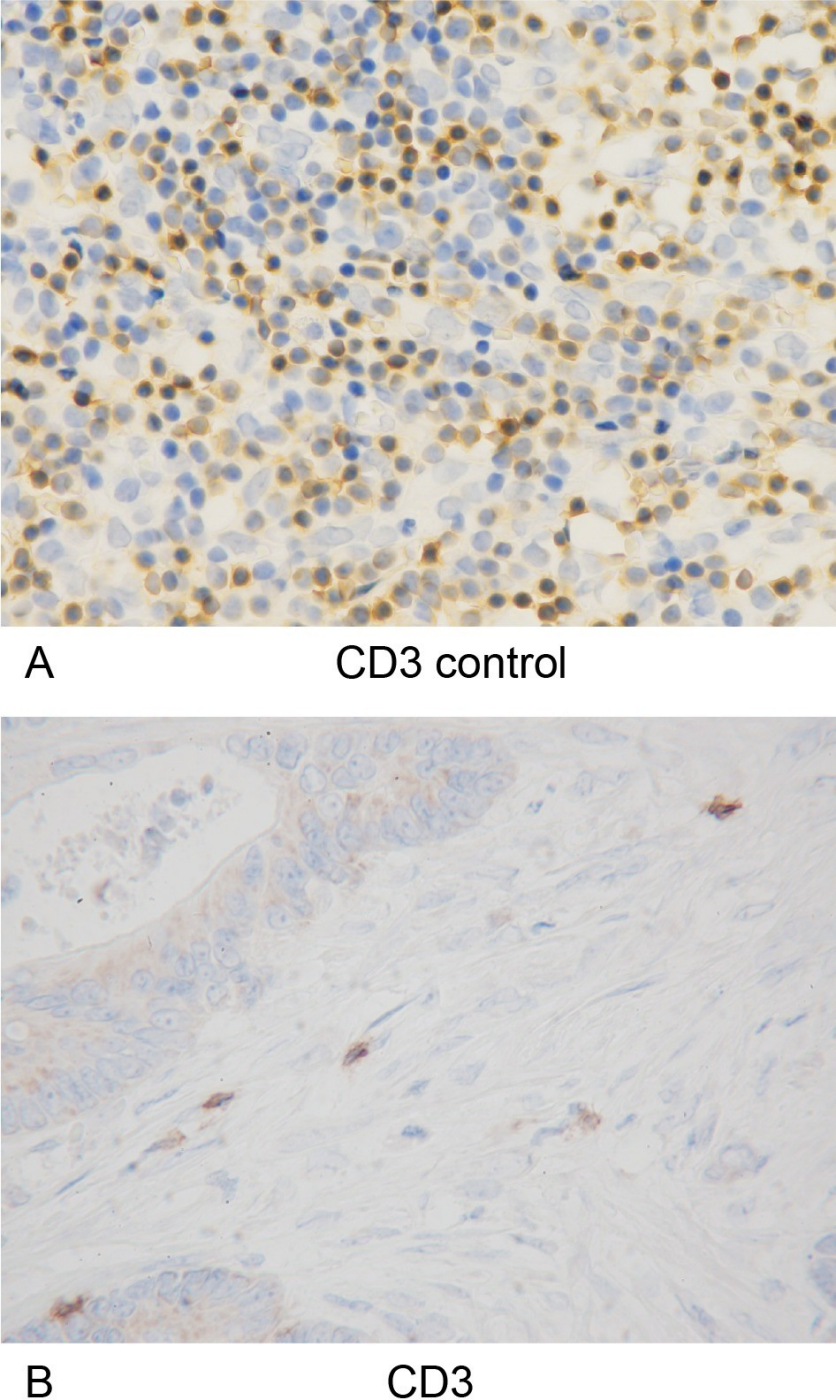
CD3-positive tumor-infiltrating lymphocytes and positive controls. (A) The specimens of lymph nodes containing CD3-positive T lymphocytes were used as positive controls for immunohistochemical staining of anti-CD3 (x400 HPF). (B) Immunohistochemical detection of CD3-positive tumor-infiltrating lymphocytes (x400 HPF).

**Fig 5:**
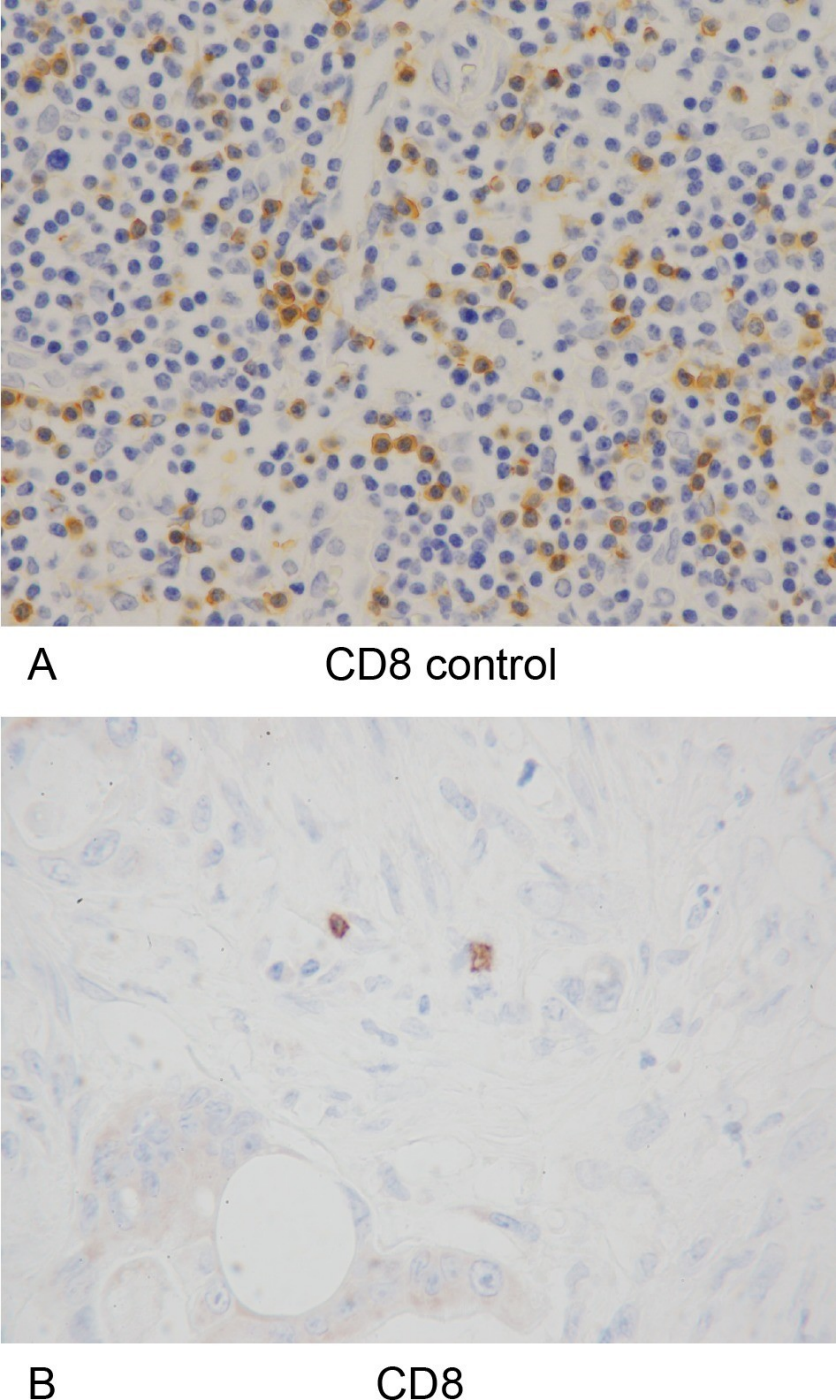
CD8-positive tumor-infiltrating lymphocytes and positive controls. (A) The specimens of lymph nodes containing CD8-positive T lymphocytes were used as positive controls for immunohistochemical staining of anti-CD8 (x400 HPF). (B) Immunohistochemical detection of CD8-positive tumor-infiltrating lymphocytes (x400 HPF).

**Fig 6:**
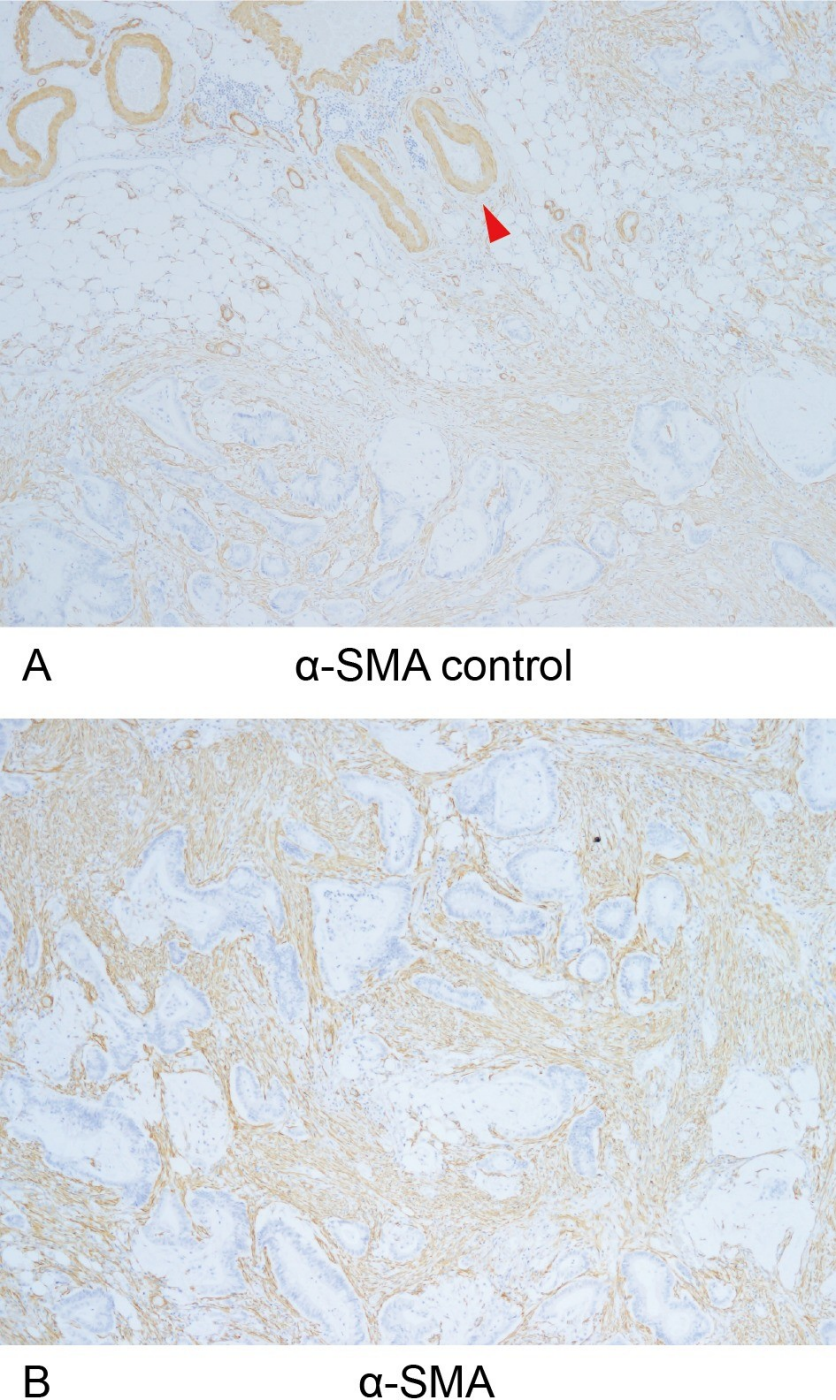
α-SMA-positive cancer-associated fibroblasts and internal positive controls. (A) α-SMA expression in vascular smooth muscle (red arrow) was used as an internal positive control for immunohistochemical staining of anti-α-SMA (x40 HPF). (B) Immunohistochemical detection of α-SMA-positive cancer-associated fibroblasts (x40 HPF).

**Fig 7:**
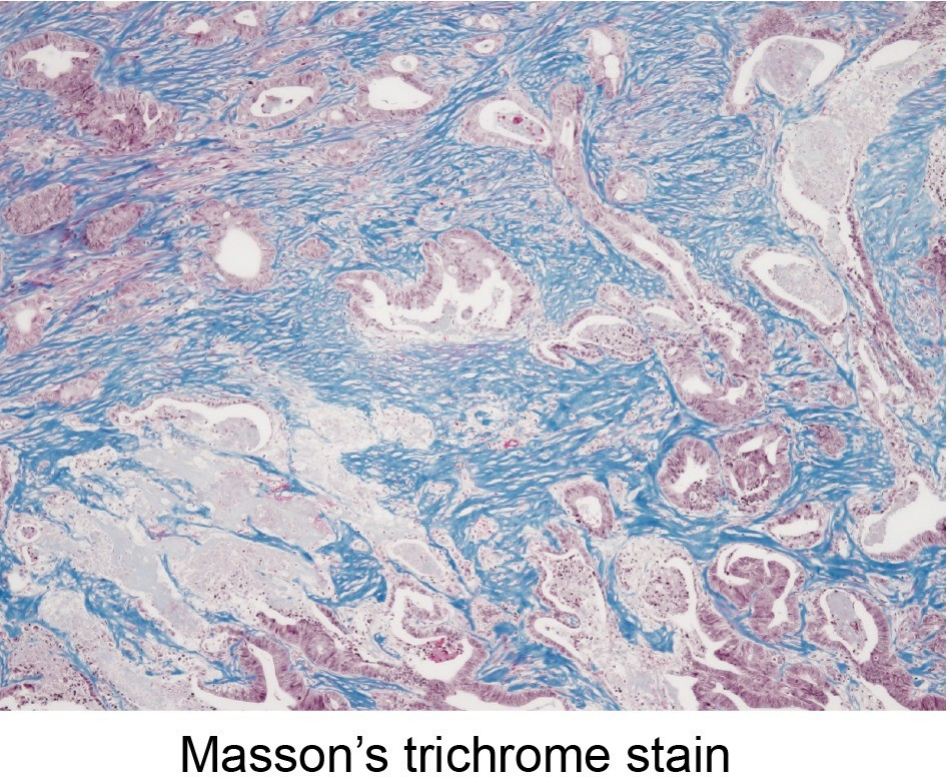
Collagen fibers. Detection of collagen fibers stained blue on Masson’s trichrome stain (x40 HPF).

**Fig 8:**
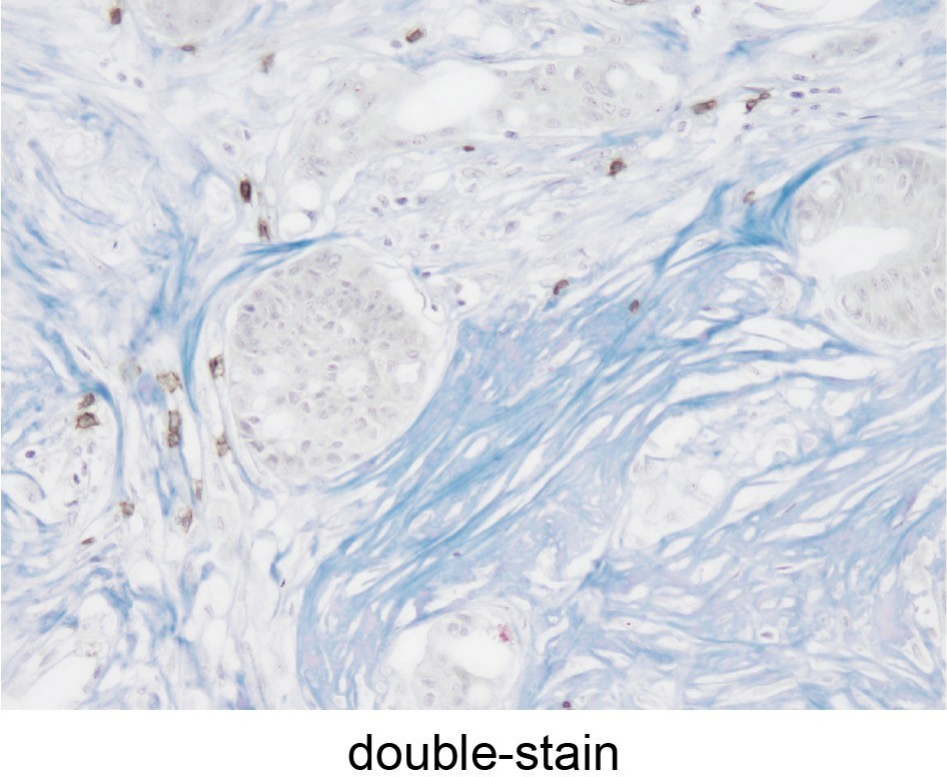
Tumor-infiltrating lymphocytes and collagen fibers using double-staining. Detection of tumor-infiltrating lymphocytes (brown) and collagen fibers (blue) double-stained using immunohistochemical staining and Masson’s trichrome staining (x200 HPF).

The number of samples of distant metastases and their primary tumors is shown in **Table 1**. Immunohistochemical staining (CD3, CD8, and α-SMA), MTS, and double staining of CD3/8 and MTS were performed, and representative images are shown, including CD3/CD8 **(Figs 4 and 5)**, α-SMA **(Fig 6)**, MTS **(Fig 7)**, and double staining **(Fig 8)**.

**Table 1:** Number of primary tumors and metastases used for the analysis.

	number of samples of metastatic tumors* (n = 230)	**number of samples of primary tumors (n = 160)**
peritoneal metastasis			67	52
liver metastasis	138	94
lung metastasis	25	14
	**number of patients (n = 218)**	
**metastases per patient for analyses**		
peritoneal metastasis only	63	
liver metastasis only	126	
lung metastasis only	17	
peritoneal & liver metastasis	4	
liver & lung metastasis	8	

*Only one sample per metastatic organ from each patient was analyzed.

### Relationship between TIL infiltration in colorectal cancer metastases and the Immunoscore in primary tumors

**Fig 9:**
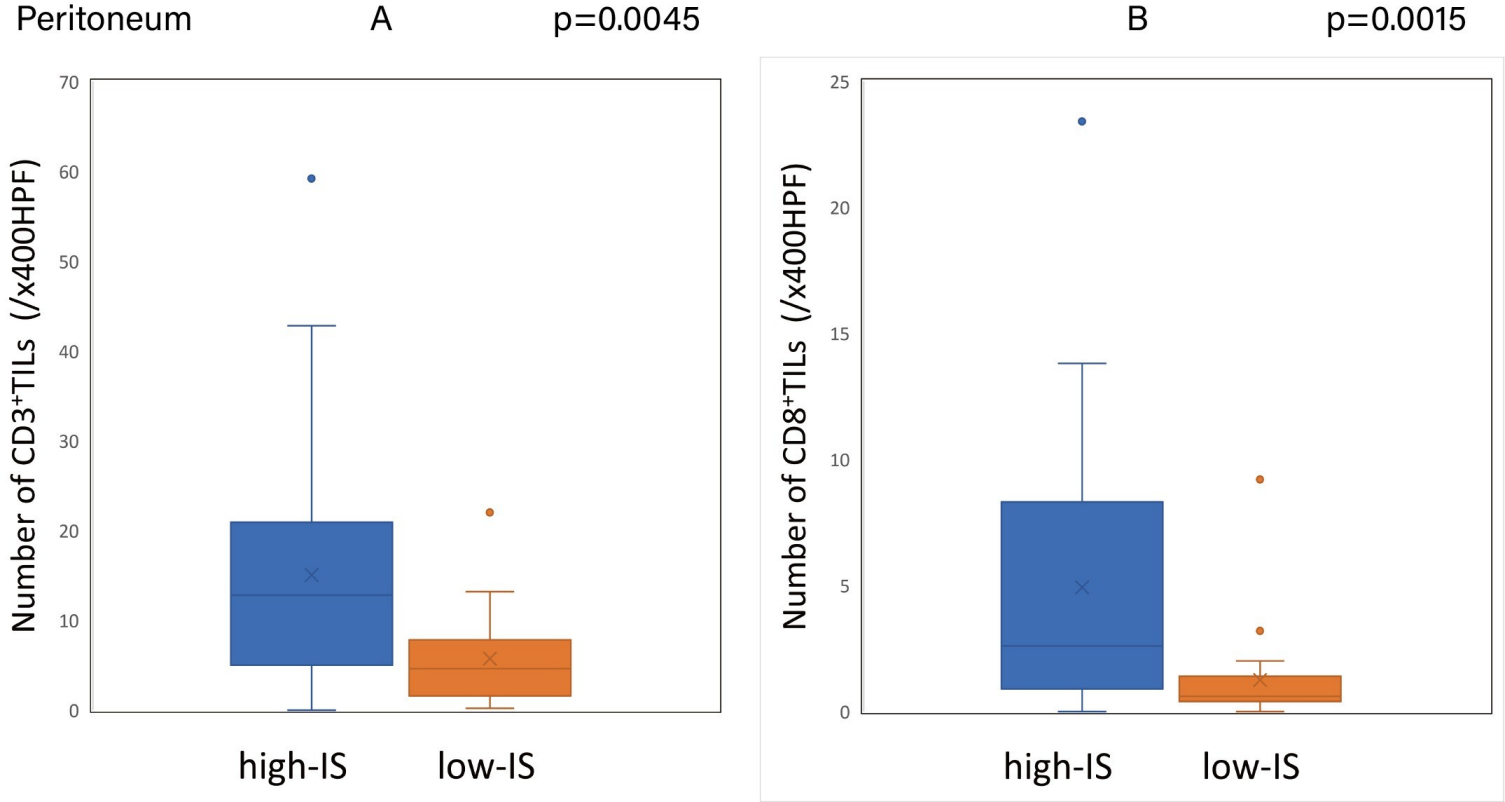
Relationship between the Immunoscore of primary tumors and the degree of TIL infiltration in the peritoneal metastases. (A) (B) The density of tumor-infiltrating lymphocytes in peritoneal metastases with high-Immunoscore primary tumors was significantly higher than that with low-Immunoscore primary tumors (CD3: p = 0.0045, CD8: p = 0.0015).

**Fig 10:**
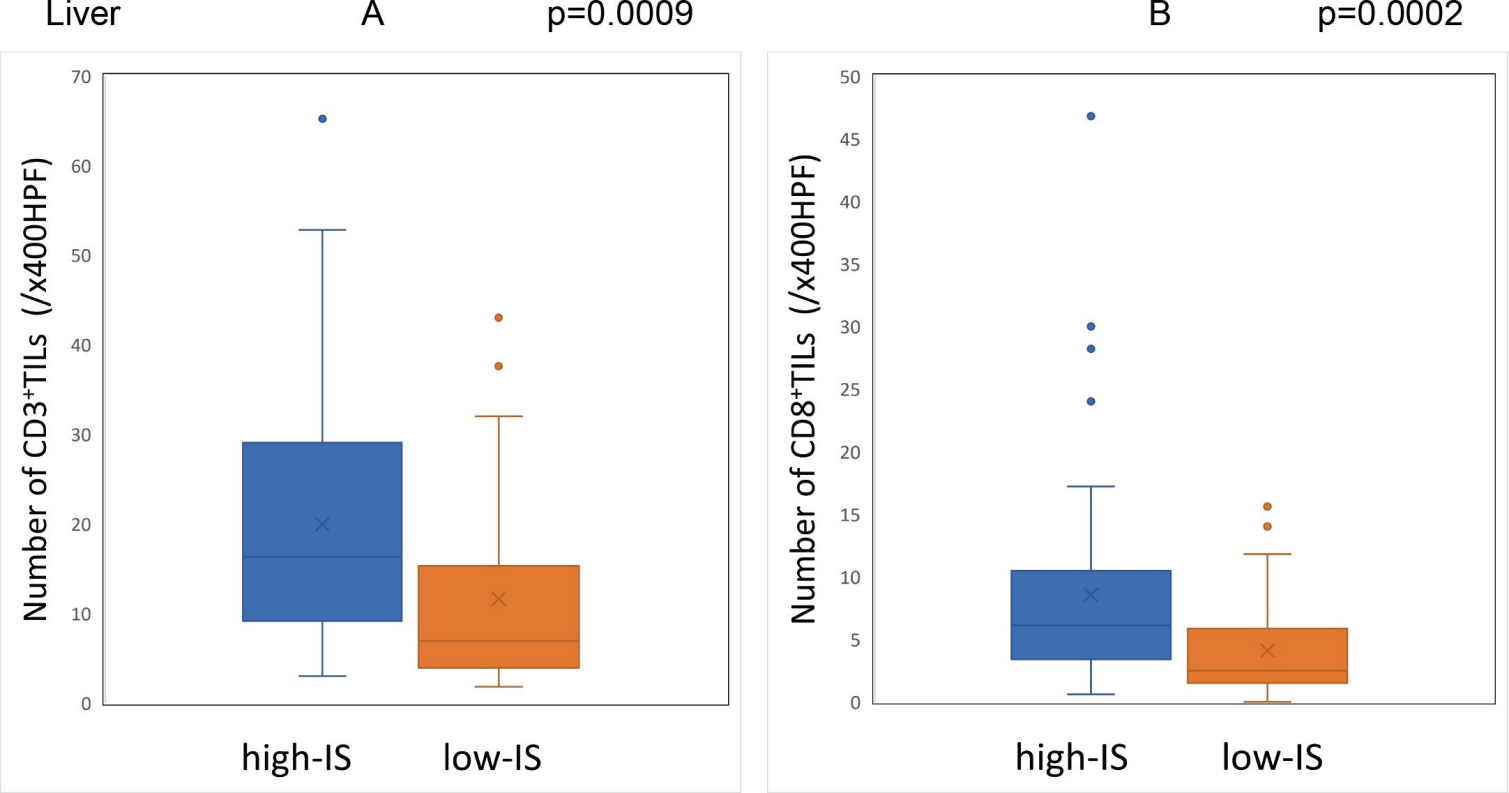
Relationship between the Immunoscore of primary tumors and the degree of TIL infiltration in the liver metastases. (A) (B) The density of tumor-infiltrating lymphocytes in liver metastases with high-Immunoscore primary tumors was significantly higher than that with low-Immunoscore primary tumors (CD3: p = 0.0009, CD8: p = 0.0002).

**Fig 11:**
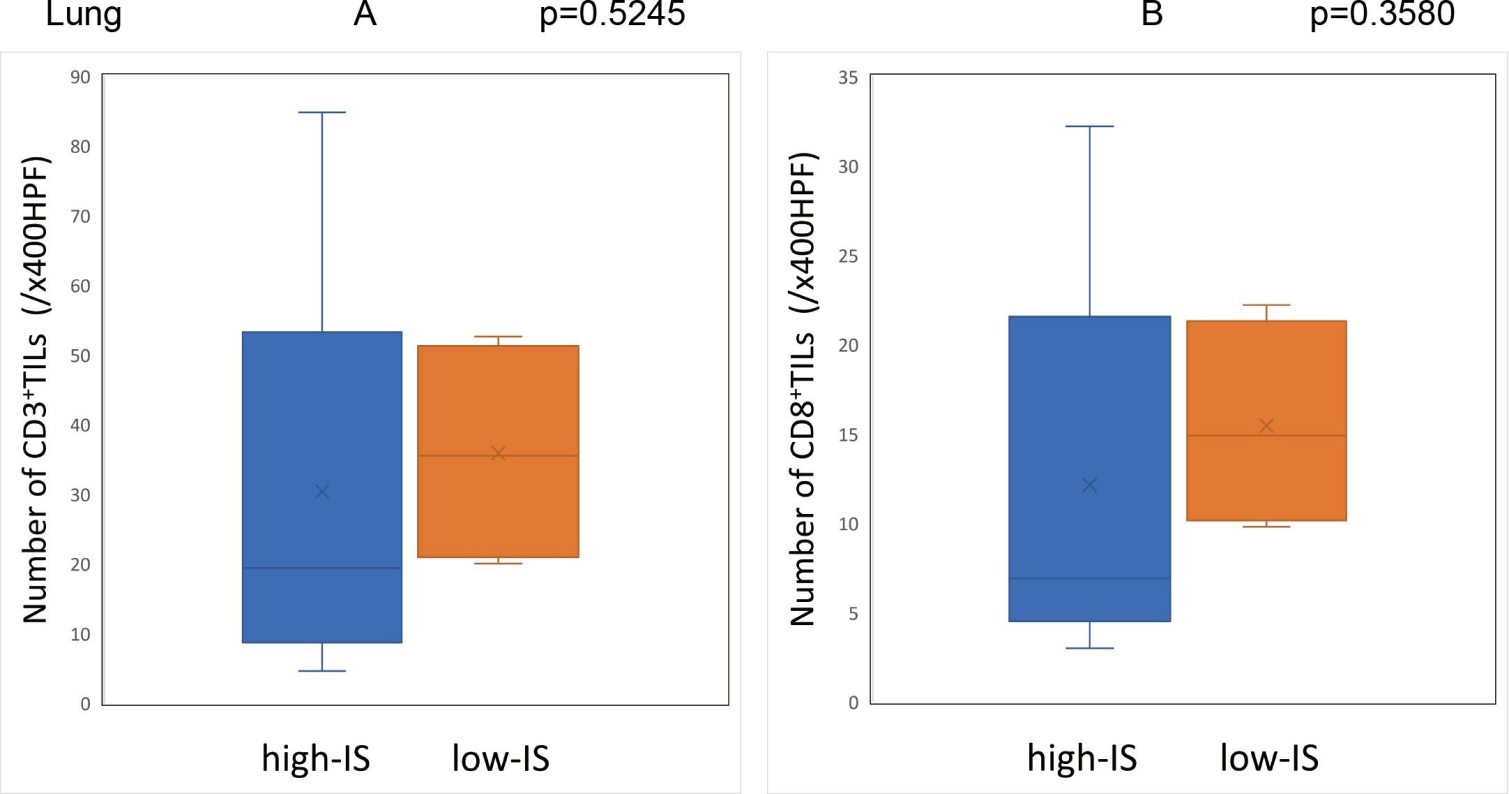
Relationship between the Immunoscore of primary tumors and the degree of TIL infiltration in the lung metastases. (A) (B) There was no significant difference between the Immunoscore of primary tumors and the density of tumor-infiltrating lymphocytes in lung metastases.

The Immunoscore was calculated using the method described above with the median densities of CD3^+^/CD8^+^ TILs in the IM and CT of the primary tumor set as the cut-off values (CD3-IM: 26.5, CD3-CT: 14.9, CD8-IM: 11.5, CD8-CT: 5.5/x400HPF). Based on the cut-off values, the patients were classified into the high-IS group with 96 cases and the low-IS group with 64 cases. The Immunoscore of the primary tumors showed a significant correlation with the degree of CD3^+^ and CD8^+^ TIL infiltration in the peritoneal and liver metastases **(Figs 9 and 10)**, although there was no correlation between the Immunoscore and the degree of TIL infiltration in lung metastases **(Fig 11) (S1 Table)**.

### The comparison of TIL infiltration and fibrosis in metastatic colorectal cancer (by organ)

**Fig 12:**
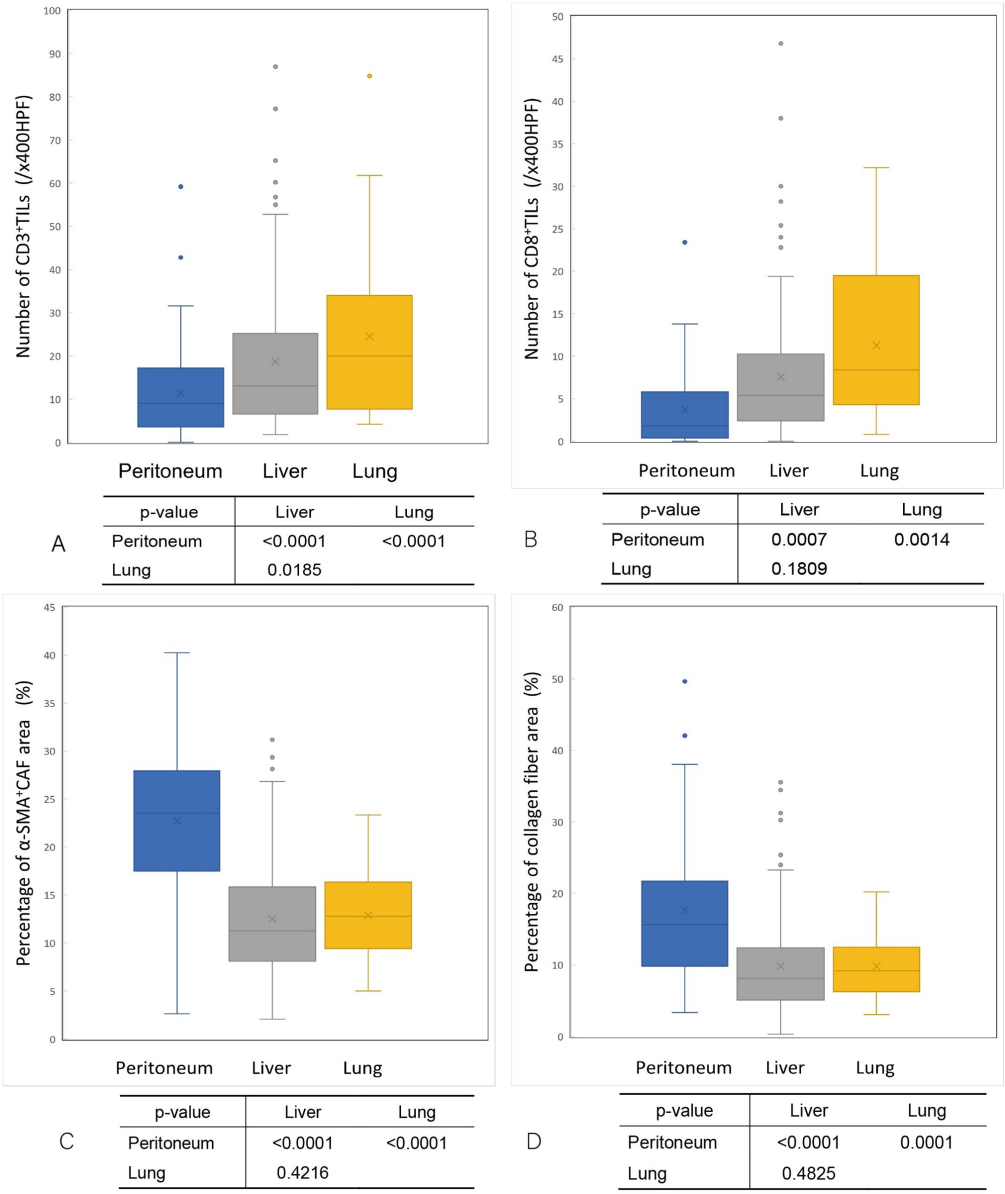
The comparison of the density of tumor-infiltrating lymphocytes and intratumoral fibrosis in each organ. (A) The density of CD3-positive tumor-infiltrating lymphocytes in peritoneal metastases was significantly lower than that in liver metastases (p<0.0001) and lung metastases (p<0.0001). (B) The density of CD8-positive tumor-infiltrating lymphocytes in peritoneal metastases was significantly lower than that in liver metastases (p = 0.0007) and lung metastases (p = 0.0014). (C) The ratio of the α-SMA-positive cancer-associated fibroblast area in peritoneal metastases was significantly greater than that in liver metastases (p<0.0001)and lung metastases (p<0.0001). (D) The ratio of the collagen fiber area in peritoneal metastases was significantly greater than that in liver metastases (p<0.0001) and lung metastases (p = 0.0001).

The density of CD3^+^TILs in peritoneal metastases was significantly lower than that in liver and lung metastases (median: peritoneal metastases 9.0 vs. liver metastases 13.1 vs. lung metastases 20.0; /x400 high-power field [HPF]) **(Fig 12A) (S2 Table)**. Similarly, the density of CD8^+^TIL infiltration in peritoneal metastases was significantly lower than that in liver and lung metastases (median: peritoneal metastases 1.8 vs. liver metastases 5.4 vs. Lung metastases 8.4; /x400HPF) **(Fig 12B) (S2 Table)**. Conversely, the ratio of the α-SMA^+^CAF area in peritoneal metastases was significantly greater than that in liver and lung metastases (median: peritoneal metastases 23.53% vs. liver metastases 11.25% vs. lung metastases 12.78%) **(Fig 12C) (S2 Table)**. Similarly, the ratio of collagen fiber area (blue on MTS) in peritoneal metastases was significantly greater than that in liver and lung metastases (median: peritoneal metastases 15.64% vs. liver metastases 8.13% vs. lung metastasis 9.18%) **(Fig 12D) (S2 Table)**.

### Relationship between the distribution of TILs and the density of fibrosis

**Fig 13:**
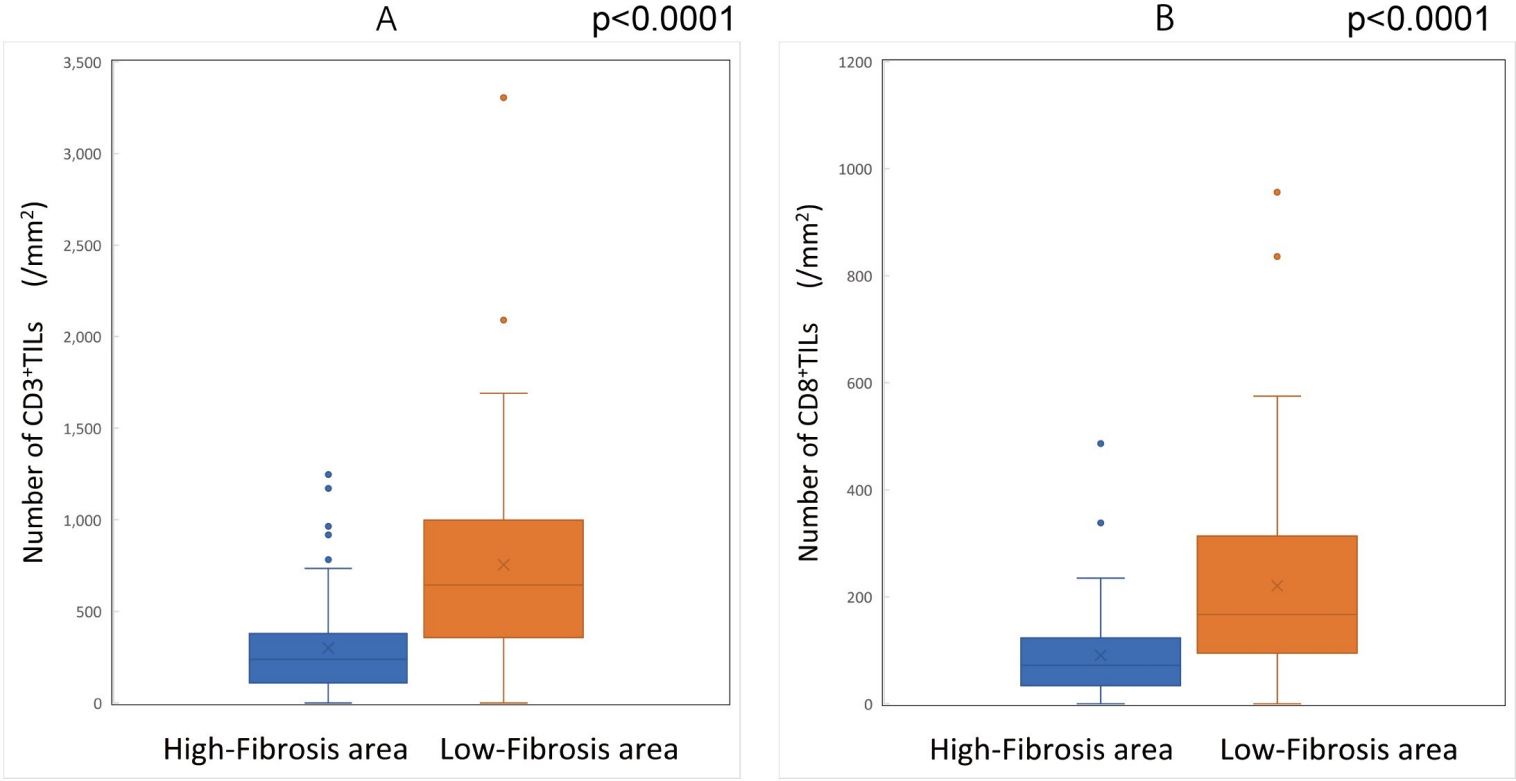
The comparison of the density of tumor-infiltrating lymphocytes in high-/low-fibrosis areas. (A) The density of CD3-positive tumor-infiltrating lymphocytes in the high-fibrosis area was significantly lower than that in the low-fibrosis area (p<0.0001). (B) The density of CD8-positive tumor-infiltrating lymphocytes in the high-fibrosis area was significantly lower than that in the low-fibrosis area (p<0.0001).

The density of CD3^+^TILs and CD8^+^TILs in the high-fibrosis area of peritoneal metastases was significantly lower than that in the low-fibrosis area **(Fig 13) (S3 Table)**.

## Discussion

The degree of TIL infiltration reportedly varies among patients [15, 16, 38], and even in the same patient, the TIL infiltration tends to differ depending on the metastatic organ. For example, TIL infiltration is relatively high in liver and lung metastases [17, 18] but relatively low in brain metastases [19]. The lungs are rich in resident immune cells because of the unique immunological environment, as the lung is constantly exposed to environmental pathogens [18]. Similarly, the liver is a major metabolic and detoxifying organ where abundant immune cells are recruited [39–41]. However, there are few TILs in the brain because inflammatory cells have difficulty reaching the brain due to the blood-brain barrier [42]. Organ-specific immunological characteristics may thus lead to differences in the TIL infiltration among organs. In the present study, we found that the degree of CD3^+^ and CD8^+^ TIL infiltration in peritoneal metastases was significantly lower than that in liver and lung metastases.

The TIL infiltration is reported to be affected by a number of factors, such as suppressive immune cells, neovascular dysfunction, suppression of chemokines, cytokines, and intratumoral fibrosis [11, 24, 25], and in the present study, we also focused on how intratumoral fibrosis affects the infiltration of TILs. We evaluated CAFs which are the main cellular component in intratumoral fibrosis, and collagen fibers which are the main non-cellular component. The results showed that there were significantly more CAFs and collagen fibers in peritoneal metastases of colorectal cancer than liver and lung metastases. By contrast, significantly fewer TILs were observed in peritoneal metastases than in liver and lung metastases. Furthermore, regarding the distribution of TILs in intratumoral fibrosis, which was observed in a micro view, the degree of TIL infiltration in the high-fibrosis area was found to be significantly lower than that in the low-fibrosis area. Given these findings, we speculated that TILs were prevented from infiltrating these tumors by the greater degree of fibrosis in peritoneal metastasis than in other organs.

Previous studies on fibrosis may explain the mechanism by which fibrosis restricts the migration of T lymphocytes [11]. First, intratumoral fibrosis prevents T lymphocytes from making contact with tumor cells by forming a physical barrier around the tumor cells. The study by Salmon et al. revealed the important role of the density of the stromal extracellular matrix in controlling the migration of T lymphocytes by real-time imaging in viable slices of lung tumors [24]. In the sparse intratumoral fibrosis area, TILs were rich, and the lymphocyte motility was high, whereas in the dense intratumoral fibrosis area, few lymphocytes were observed, and the motility was also decreased. Second, CAFs exclude T lymphocytes via CXCL12/CXCR4 [11, 43]. The chemokine CXCL12 released from tumor cells and CAFs may suppress the infiltration of T lymphocytes through a mechanism called chemorepulsion mediated by CXCR4, which is the receptor for CXCL12 and also expressed on T lymphocytes [27, 44, 45]. Third, high interstitial fluid pressure restricts the migration of T lymphocytes to tumor cells. High intratumoral fibrosis reportedly results in high interstitial fluid pressure in tumors [46]. An increased interstitial fluid pressure impedes the infiltration of TILs into the tumor because the migration of T lymphocytes from the tumor blood vessels into the stroma must overcome a high pressure difference [47].

As for why intratumoral fibrosis is high in peritoneal metastasis, we speculated that tumor cells detached from the primary tumor into the peritoneal cavity, attached to the peritoneal mesothelial cells, and invaded the subepithelial zone where fibroblasts accumulate to provide a microenvironment that supports the establishment and progression of peritoneal metastasis [48–51]. It was thought that fibrosis was high in peritoneal metastases because metastases formed from the subepithelial stroma, where fibroblasts are abundant [28, 48, 50].

Several basic studies on treatments that target fibrosis to activate immunity have been reported. In the study by Salmon et al., collagenase was used to reduce collagen fibers in the tumor stroma, thereby enhancing T lymphocytes infiltration. In the study by Wen et al., a DNA-based vaccine targeting fibroblast activation protein (FAP) in CAFs was designed to immunize model mice of colon cancer, resulting in the decreased expression of FAP^+^CAF and collagen fibers in the tumor stroma, thereby leading to an increase in T lymphocyte infiltration. As a result, the formation of primary tumors and the growth of metastatic tumors was significantly suppressed [52]. Furthermore, Feig et al. reported that, in a mouse model of pancreatic ductal adenocarcinoma, the administration of an inhibitor against CXCR4, which has been reported to be involved in the suppression of TIL infiltration, induced the rapid accumulation of T lymphocytes in tumors, resulting in a reduction in cancer cell numbers [53]. Based on these findings, the clinical application of treatments targeting fibrosis may promote T lymphocyte infiltration and improve the prognosis of patients with peritoneal metastasis.

In this study, we compared the local immunity of primary tumors with that of distant metastases. Since the local immunity of both the primary lesion and the metastases reflects the systemic immunity of the host, the degree of TIL infiltration in the primary tumors was presumed to correlate with that in the metastases. The results obtained in this study support this speculation, showing a significant correlation between the Immunoscore of primary tumors and the degree of TIL infiltration in peritoneal and liver metastases. However, the degree of TIL infiltration in distant metastases is greatly influenced by organ-specific characteristics, such as fibrosis, which may be one reason why the therapeutic outcomes vary among metastatic organs.

Several limitations associated with the present study warrant mention. First, this study was a retrospective, single-center study with a relatively small number of patients. Second, only fibrosis was examined as a factor affecting the infiltration of TILs in this study; however, the infiltration of TILs is regulated by the comprehensive activities of various factors, such as immunocompetent cells, including tumor-associated macrophages, dendritic cells, and myeloid-derived suppressor cells, as well as the chemokines involved in the recruitment of tumor antigen-presenting cells and immunocompetent cells [11, 25].

## Conclusion

The present findings suggest that the degree of TIL infiltration in distant metastases correlates with that in primary tumors of colorectal cancer but is greatly influenced by organ-specific characteristics. In addition, in cases of colorectal cancer, there is more fibrosis and less TIL infiltration with peritoneal metastases than with liver and lung metastases. Furthermore, by investigating the local distribution of microenvironmental components in tumors, we found that the infiltration of TILs was poor in areas where fibrosis was high. A high level of fibrosis in peritoneal metastases of colorectal cancer restricts the infiltration of T lymphocytes into the tumor and may be a factor that leads to treatment resistance and a poor prognosis.

## Supporting information

S1 Table Relationship between the Immunoscore of primary tumor and the degree of TILs in the metastases. (XLSX)

S2 Table The comparison of TIL infiltration and fibrosis in metastatic colorectal cancer. (XLSX)

S3 Table Relationship between the density of TILs in high-fibrosis area and that in low-fibrosis area. (XLSX)
